# Connecting Care Closer to Home: Evaluation of a Regional Motor Neurone Disease Multidisciplinary Clinic

**DOI:** 10.3390/healthcare13070801

**Published:** 2025-04-02

**Authors:** Karen Hutchinson, Georgia Fisher, Anna Schutz, Sally Carr, Sophie Heard, Molly Reynolds, Nicholas Goodwin, Anne Hogden

**Affiliations:** 1 Centre for Healthcare Resilience and Implementation Science, Australian Institute of Health Innovation, Macquarie University, Sydney, NSW 2109, Australia; georgia.fisher@mq.edu.au; 2 Central Coast Local Health District, Gosford, NSW 2250, Australia; anna.schutz@health.nsw.gov.au (A.S.); sally.carr@health.nsw.gov.au (S.C.); sophie.heard@health.nsw.gov.au (S.H.); nick.goodwin@health.nsw.gov.au (N.G.); 3 Central Coast Research Institute, University of Newcastle, Gosford, NSW 2250, Australia; 4 School of Population Health, Faculty of Medicine and Health, University of New South Wales, Sydney, NSW 2052, Australia; a.hogden@unsw.edu.au; 5 School of Medicine, Faculty of Medicine and Health, University of Sydney, Sydney, NSW 2006, Australia; 6 Department of Neurology, Concord Hospital, Sydney Local Health District, Sydney, NSW 2139, Australia

**Keywords:** motor neurone disease, multidisciplinary clinic, implementation science, behaviour change, barriers, facilitators, regional health services

## Abstract

The optimal approach to managing motor neurone disease (MND) is through integrated, person-centred care (PCC), complemented by access to specialised MND multidisciplinary clinics (MDCs). However, in Australia, MND care is fragmented and uncoordinated. Objectives: To evaluate participant experiences of the implementation of a new regional MND MDC in New South Wales, Australia, and explore factors influencing its implementation. Methods: A qualitative evaluation was conducted. We used semi-structured interviews with people living with MND (plwMND) (*n* = 4), family carers (*n* = 2), healthcare providers (*n* = 6), and social care providers (*n* = 2). First, deductive analysis using the Theoretical Domains Framework and COM-B model was applied to identify factors influencing the adoption and sustainability of the MDC. Then, an inductive thematic analysis identified the impact of the MND MDC from participant perspectives. Results: The MND MDC was found to be appropriate and acceptable for providing equitable access to PCC MND care that was ‘*closer to home*’. The three main themes from the inductive analysis indicated that: 1. Implementing it was a ‘*good idea*’ [the MND-MDC]; 2. It ‘*flushes out*’ local service gaps and/or challenges; and 3. It results in positive outcomes. Key facilitators to implementation identified from the deductive analysis were staff expertise, strong trusting relationships with all clinic participants, and the belief that the MND MDC optimised care. Barriers to implementation included a lack of staff remuneration, organisational boundaries, limited representation of specialities, and anticipated difficulties in coordinating care with existing services. Conclusions: The commitment to providing equitable access to an MND MDC in a regional area is crucial to optimise care for plwMND and their families. However, overcoming complex organisational boundaries, creating local solutions, and building strong partnerships are key challenges to securing ongoing financial support and local health district ‘buy-in’ to support sustainability.

## 1. Introduction

Motor neurone disease (MND) is a rare, progressive neurological condition with no known cure, perpetuating a considerable burden of disease and economic impact. As MND progresses, so too does its impact on the everyday function of people living with the condition (plwMND) and their families, increasing their need for support from health and social care and for complex medical and assistive equipment [1,2]. The best available evidence indicates that plwMND should receive person- and family-centred care that is delivered in coordinated specialised multidisciplinary clinics (MND MDCs) by expert clinicians [3,4]. Such specialist care can extend survival and improve the quality of life of plwMND and their family and carers [4,5,6,7,8]. Additionally, MND MDC clinics increase the speed of access to essential interventions for plwMND, which is critical to their improved survival [2,4,9].

### 1.1. The Australian Context

Most specialist MND MDCs in Australia [10] are based across six metropolitan areas and vary widely in the way they operate, are resourced, and are integrated into the local health and community networks where plwMND reside [5,11,12]. Thus, plwMND who reside outside metropolitan centres in regional and rural areas face multiple barriers, a fact echoed in research from Canada [13], Ireland [6], and England [14], in attending MDCs and receiving timely and coordinated care that could prolong both the quality of their life and their life expectancy [6,13,15]. Globally, several countries have developed clinical pathways and guidelines that encourage collaboration between specialist MND- MDCs and recommend that a vast array of health and social care providers support plwMND and their families in their local communities [3,16,17]. However, such guidelines are absent in the Australian context, where the transition of care, communication, and support between specialised MND MDCs and health and social care providers remains fragmented and limited in many parts of the country [4,5]. In Australia, integration of care varies both across and within states and territories due to differences in governance and funding allocation, the lack of shared medical records, large geographical distances, and cross-border logistical and legislative challenges [18,19]. While all Australian citizens have universal access to low- or no-cost healthcare via Medicare [20], access to disability and aged care is firmly determined by someone’s age. The National Disability Insurance Scheme (NDIS) [21] provides non-means-tested funding to eligible plwMND < 65 years, whereas My Aged Care [22] provides means-tested funding to plwMND > 65 years. These funding schemes complicate collaboration amongst care providers and are based on a marketized system that has resulted in care fragmentation, and only limited resources are available for communication between care providers to facilitate knowledge exchange and care coordination [23,24,25].

### 1.2. Integrated Models of Care

Delivering a person-centred integrated care model to ensure plwMND receive the right care in the right place at the right time is a potential solution to the issues with MND care in Australia [26]. Integrated care has two key components, the unification of elements of care provision and information sharing, which are fragmented in both design and delivery, and the delivery of high-quality and safe health and/or social care through continual and ‘co-productive partnerships’ [27] (p. 2) to a target population [11,28,29]. Integrated care improves communication between sectors and organisations and also between healthcare (HCP) and social care professionals (SCP), healthcare consumers, and their carers and families. Ultimately, this promotes seamless and coordinated healthcare pathways [29,30,31], improving the experiences of both people living with chronic and complex conditions and the people who care for them [4,28].

In MND care, adopting integrated approaches to care can potentially overcome the current roadblocks to care coordination across MND MDCs, health and social care, and aged and disability care providers [4,32,33]. A review of the international literature (unpublished) indicated that integrating care can improve patient survival time [34] by promoting timely non-invasive ventilation and gastrostomy use, which can be supported with ongoing monitoring in the community [4,7]. Moreover, improved quality of life is evident where high standards of care improve mental health and wellbeing, with timely access to care and support from diagnosis to end of life [5,15] There was an enhanced response as the care needs and priorities of plwMND and their carers can change with the stage and duration of MND, necessitating the greater integration of care and support provision [6,35]. Finally, integrated MND MDCs supporting complex shared decision-making with plwMND and family can also support and improve the knowledge of community-based health and social care providers [33,36].

Thus, a regionally based, integrated MND MDC has the potential to provide plwMND in regional areas of Australia with equitable access to coordinated and connected care. We established one such clinic on the Central Coast, NSW, Australia. The Central Coast has an area of 1680 km^2^ and a population of 346,596 according to the 2021 Australian Bureau of Statistics census [37]. The MND MDC was designed to provide care coordination and clinical expertise and facilitate referral pathways for plwMND and carers to access timely care and support in their local area. Whilst the evidence on the importance of specialist MND MDCs is clear [4,5], there is very little information available on the optimal organisational structure of such a clinic and how it might best function outside of metropolitan areas.

Therefore, this study aimed to evaluate participant experiences of a regional MND MDC on the Central Coast, NSW, Australia, and explore the factors influencing MND MDC implementation.

## 2. Materials and Methods

### 2.1. Ethics Approval

This study received approval from the Northern Sydney Local Health District Human Research Ethics Committee (HREC: 2022/ETH00142).

### 2.2. Intervention

**Figure 1:**
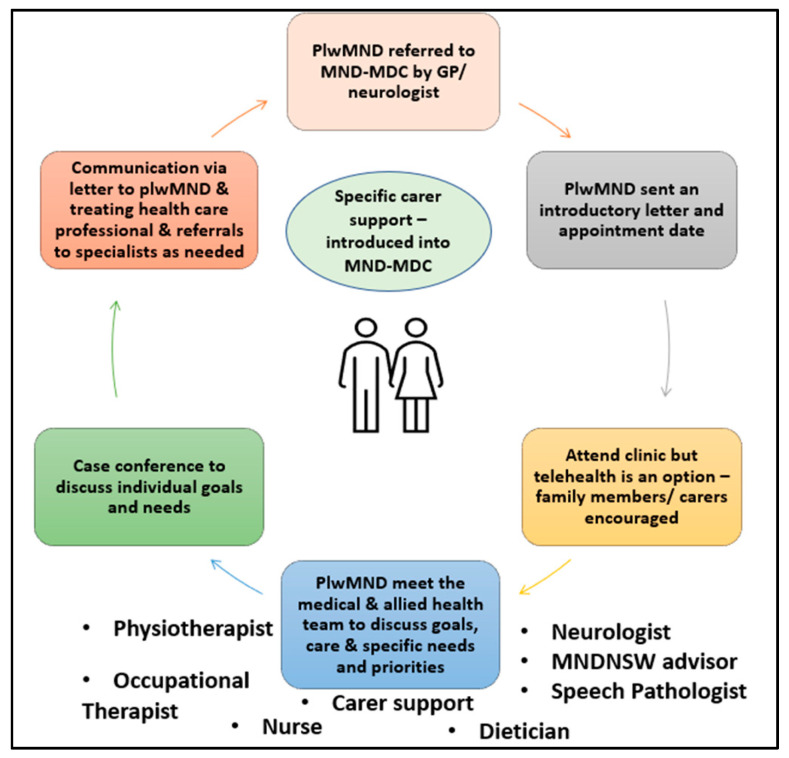
Overview of the MND clinic’s operation pathway.

An MND MDC clinic was established for plwMND and their carers on the Central Coast, NSW, and commenced services in February 2020 (Figure 1). The clinic was held in a private neurology clinic, but there were no clinic and/or consultant fees for plwMND. As with many innovations in health service delivery, the MND MDC clinic was based on a ‘good idea’ that spread through ‘informal, decentralised, horizontal social networks’ [38] (p. 597). Based on feedback from plwMND, carers, and local healthcare professionals, the clinic neurologist identified the need for the MND MDC and then used their professional network to recruit locally based HCPs and SCPs with expertise in MND care. HCPs were required to attend the clinic in person, to have the ability to work as private practitioners, and possess knowledge of the local health service context. Professionals employed in the publicly funded health workforce could only attend the MND MDC remotely due to legislative restrictions on those working in publicly funded health services.

The clinic was held once every four months and led by the founding neurologist, who worked in the clinic alongside a physiotherapist, an occupational therapist, a speech pathologist, a dietician, a nurse, a carer support officer, and a representative from MND NSW, a state-wide non-profit organisation that supports plwMND. The clinic also supported teleconference access to a palliative care specialist, MND NSW Association representatives, and very occasionally to a respiratory physician. Some clinic staff that worked in private practice had professional connections to the publicly funded local health district (LHD) and so acted as a link between the private and public sectors.

### 2.3. Study Design

This study was a qualitative multi-method evaluation of a regionally based MND MDC from the perspectives of plwMND, family carers, and clinic staff. This research design was used to capture a rich and detailed understanding of the experiences and perceptions of all who participated in the implementation of the MND MDC [39].

Interviews were conducted, and two methods of qualitative analysis were applied to the interview data. Sequential deductive and inductive analysis were used to generate a deep and nuanced interpretation of the data and allowed us to understand how well the clinic was perceived by participants, and why this was important to them [40]. This has particular importance for gaining perspectives and experiences from mixed groups of participants [39,41,42].

This research design facilitated an understanding of current practices, procedures, challenges, and the level of integration into the health and social care system to better understand the MND MDC’s impact on managing MND in a regional setting and factors influencing its implementation.

### 2.4. Study Development and Guidance

The study was co-designed by a team of researchers with experience in integrated care, implementation science, and MND, along with Central Coast Local Health District (CCLHD) clinicians with expertise in the management of MND. A stakeholder advisory group (SAG) was also formed to ensure relevancy to the local context and those delivering, receiving, and managing the service, and to provide study guidance and credibility to the evaluation [43]. The SAG comprised twenty members: plwMND, carers, the MND MDC’s neurologist and palliative care specialist, nurse specialists (respiratory, neurology, and palliative care), a mix of MND MDC and non-MND-MDC allied health professionals (from both private and public sectors), health managers, research team members, MND NSW association representatives, and CCLHD Carers Support Unit representatives.

During data analysis, the SAG members sought to clarify and explore topics and themes arising from the interviews to ensure the accuracy and trustworthiness of the analysis and future MND MDC priorities [43]. The SAG met three times with the research team throughout this study. The group met through MS Teams videoconferencing for approximately one hour.

### 2.5. Data Collection

We conducted one-on-one, semi-structured interviews with plwMND and family carers who attended the MND MDC, as well as HCPs and SCPs who worked at the MND MDC. All data were collected before being analysed sequentially.

An interview schedule [Supplementary Materials File S1] was developed using open-ended questions that allowed for responses to cover the domains of the TDF, and additional probing questions ensured that contextual detail and a more open narrative were captured [44]. Semi-structured interviews were conducted by three experienced clinician-researchers (KH, MR, and SH) who worked routinely with HCPs, SCPs, and people living with or affected by MND. The interviews ranged from 30 to 60 min due to clinic staff availability and the fatigue of plwMND. They were conducted using MS Teams videoconferencing or in person, according to individual preferences, and audio was recorded using a digital recorder. One participant was unable to communicate verbally; therefore, the interview was transcribed in real time by the interviewer based on the participant’s written responses to the interview questions. Field notes were taken at each interview to record observations, experiences, and insights to complement the interview data. Similar insights and responses to questions amongst the study participants demonstrated sufficient information power relevant to this study’s aim [45]. All interviews and field notes were de-identified by a researcher (KH) and given a code name according to their characteristics (e.g., HCP or plwMND), and a professional organisation transcribed the interviews.

### 2.6. Recruitment

Purposive sampling [46] was used to recruit participants who had experience with the MND MDC, either as a staff member or as an attendee—a plwMND or an informal/family carer of an attendee. HCPs and SCPs, as well as plwMND and their family carers, were excluded if they had not participated in the implementation of the MND MDC. The lead researchers (AS and KH) at the MND MDC invited all 15 plwMND and carers who attended the clinics between June and October 2022 to join this study. All HCP and SCP staff at the MND MDC were invited to participate by the same lead researchers. Participants were provided verbal information about the research regarding the MND MDC and a targeted participant information and consent form. Participants who agreed to be interviewed provided written consent prior to data collection.

### 2.7. Analysis

A two-stage sequential analysis of deductive and then inductive analysis was undertaken. This hybrid approach was chosen due to the nascent evidence on MND MDCs to ensure that a broad range of influential factors and the outcomes of the implementation were captured [44].

#### 2.7.1. Deductive Analysis

**Figure 2:**
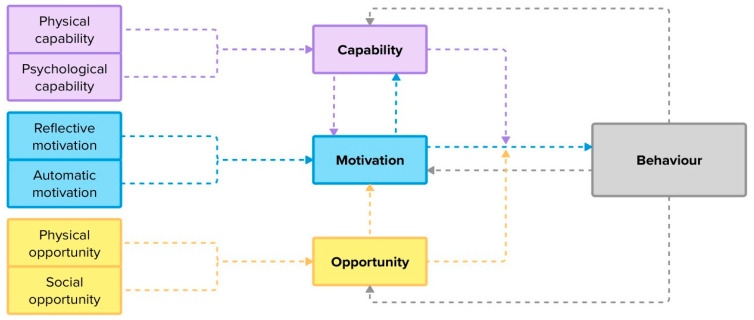
COM-B model of behaviour change. Arrows in the model represent the influence of one component on another. Note that opportunity and capability influence the relationship between motivation and behaviour, rather than the components individually. Figure adapted from Michie et al. (2011) [48].

First, we applied the TDF [47] and the COM-B [48] to the interview data (Table 1 and Figure 2). The TDF was originally designed to be used with HCPs but was adapted in this analysis to be concurrently used with ‘patient/public population groups’ [44] (p. 690). The COM-B (Figure 2) captures an individual’s **capabilities** (can I do this?), **opportunities** (is it possible to do this?), and **motivations** (do I want to do it?) to engage in a target behaviour [48,49]. The TDF builds on the COM-B (Table 1) to categorise drivers of behaviour change more explicitly across 14 domains, from both a service user and provider perspective, and informs appropriately targeted strategies to help adopt change [47,49,50]. Together, these tools are widely used in tandem to explain contextual influences on behaviour when implementing practice change [47]. This created a coding frame to identify behavioural determinants of sustainable engagement with the MND MDC [47,51].

After reading all transcripts to familiarise themselves with the data, two researchers (GF and KH) independently extracted and coded the interview data using a mutually agreed guideline. Interview data were first categorised according to the 14 domains of the TDF and then mapped to the COM-B model (see Table 1 and Figure 2) to identify barriers to and facilitators for sustainable engagement with the MND MDC [47,51]. Once both researchers had coded all data, they met to resolve any disagreements, and similar beliefs were grouped together to develop belief statements, i.e., “a collection of responses with a similar underlying belief” [47] (p. 12), [52]. The transcript excerpts were then re-read to ensure that the belief statements accurately reflected the shared meaning across the interview data. The relevant TDF domains associated with the belief statements were identified through discussions between the two researchers and confirmed by a third researcher (AH) until consensus was reached [53]. Finally, the same two researchers mapped the belief statements to the associated components of the COM-B model and used the model to hypothesize relationships between the belief statements (Figure 2).

**Table 1:** Theoretical Domains Framework and associated components of the COM-B model [50,54,55]. Table adapted from Atkins et al. 2017 [47].

TDF Domain	Definition	COM-B Component
1. Knowledge	An awareness of the existence of something	Psychological capability
2. Skills	An ability or proficiency acquired through practice	Physical capability
3. Social/professional role and identity	A coherent set of behaviours and displayed personal qualities of an individual in a social or work setting	Reflective motivation
4. Beliefs about capabilities	Acceptance of the truth, reality, or validity about an ability, talent, or facility that a person can put to constructive use	Reflective motivation
5. Optimism	The confidence that things will happen for the best or that desired goals will be attained	Reflective motivation
6. Beliefs about Consequences	Acceptance of the truth, reality, or validity about outcomes of a behaviour in a given situation	Reflective motivation
7. Reinforcement	Increasing the probability of a response by arranging a dependent relationship, or contingency, between the response and a given stimulus	Automatic motivation
8. Intentions	A conscious decision to perform a behaviour or a resolve to act in a certain way	Reflective motivation
9. Goals	Mental representations of outcomes or end states that an individual wants to achieve	Reflective motivation
10. Memory, attention, and decision processes	The ability to retain information, focus selectively on aspects of the environment, and choose between two or more alternatives	Psychological Capability
11. Environmental context and resources	Any circumstance of a person’s situation or environment that discourages or encourages the development of skills and abilities, independence, social competence, and adaptive behaviour	Physical opportunity
12. Social influences	Those interpersonal processes that can cause individuals to change their thoughts, feelings, or behaviours	Social opportunity
13. Emotion	A complex reaction pattern, involving experiential, behavioural, and physiological elements, by which the individual attempts to deal with a personally significant matter or event	Automatic motivation
14. Behavioural regulation	Anything aimed at managing or changing objectively observed or measured actions	Psychological capability

**Figure 3:**
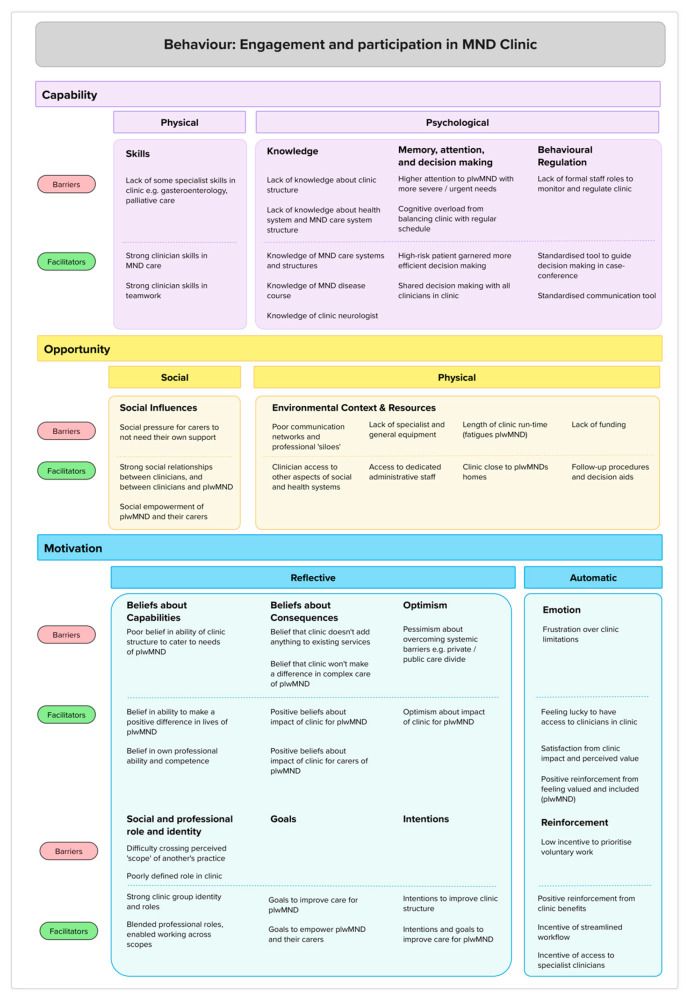
Key barriers and facilitators in each domain of the Theoretical Domains Framework mapped to each component of the COM-B model of behaviour. (Barriers and facilitators run horizontally in rows, while TDF components are divided into columns).

Footnote: The colours are used to link capability, motivation and opportunity across Table 1 and Figure 2 and Figure 3.

#### 2.7.2. Inductive Analysis

Inductive analysis was then undertaken on the interview data. Using reflexive thematic analysis [41], we aimed to identify the outcomes of the clinic and the associated nuance and context [39,44], along with any barriers to and facilitators of implementation that were additional to the TDF domains. Analysis began with familiarisation of the interview dataset [39]. Four research team members, including two clinicians, a clinician-researcher, and a researcher (KH, AH, MR, and SH), analysed the same two de-identified interview transcripts to develop a consensus on coding before independently coding a portion of the remaining interview transcripts. Codes were synthesised through collaborative and reflexive discussions between the four research team members, and a framework was iteratively developed for coding all the interviews to identify relationships and inform the generation of the main themes and sub-themes (see Table 2 [41]).

## 3. Results

Fourteen people participated in the study interviews, comprising HCPs, SCPs, plwMND, and family carers (Table 3 and Table 4). The HCPs comprised a neurologist, a palliative care specialist, a dietician, a physiotherapist, an occupational therapist, and a speech pathologist. The SCPs comprised representatives from two support organisations. All HCPs and SCPs were female.

Due to the small number of staff at the MND MDC, participant characteristics were limited to the employment sector rather than individual professional roles to avoid compromising participant confidentiality.

### 3.1. Results of the Deductive Analysis

The behavioural determinants of the capability, opportunity, and motivation to be involved in the MND MDC emerged from the deductive analysis. Figure 3 outlines the key barriers and facilitators in each domain of the Theoretical Domains Framework mapped to each component of the COM-B model for behaviour change.

#### 3.1.1. Behavioural Determinants of Capability to Be Involved in the Clinic

There was an even mix of barriers and facilitators regarding the participants’ capability to be involved in the clinic. A commonly reported facilitator was excellent knowledge of the MND care system and structure; however, this was detracted from by an overall lack of knowledge about the structure of the health system and how to navigate it effectively, as well as about the structure and purpose of the clinic itself. For example, ‘*It [the health system] can be pretty hard to navigate, who do you talk to and who do I liaise with?*’ (HCP7). Similarly, most participants recognised that the clinicians working in the clinic had strong skills in their respective areas of MND care, which facilitated the function of the clinic ‘*Now, they [clinic staff] all, work with people with MND, have had years of experience working with people with MND*’ (HCP3). The positive impact of the clinicians’ skills was somewhat hampered by a lack of consistent representation of some skills in the clinic, for example, those provided by palliative care, respiratory, and gastroenterology specialists, e.g., ‘*we have tried to get rehab and respiratory involved, but that has not been successful to date*’ (HCP6).

#### 3.1.2. Behavioural Determinants of Opportunity to Be Involved in the Clinic

The social opportunity to participate in the clinic was overwhelmingly facilitated by the strong relationships between clinic staff members, and between staff and plwMND and their carers, e.g., ‘…*it’s [the clinic] much more like a friendly—it’s much more friendly and more comfortable. And you feel more at home than a clinical situation, like, in the other places I’ve been to*’ (plwMND2). These social relationships created a warm and inviting environment, which improved the flow of the clinic, its ability to deliver person-centred care, and the sense of openness of the clinic for participants. There were also strong facilitators of the physical opportunity to participate in the clinic as its location was close to home and easily accessible by most participants. For example:

*‘So, [the clinic] is pretty central within Central Coast. Yeah, I just think instead of later when you are in that stage being in a wheelchair and stuff like that trying to get in and out [of car] and an hour and a half [travel] down into the Sydney and it’s a lot more. It’s definitely a lot more tiring and fatiguing [going to Sydney]’.*

(plwMND1)

In addition, there was access to administrative staff that made it easier for all participants to engage in the clinic, ‘*The admin staff are brilliant. Whenever we need to, I can ring and get [work specific] certificates [to attend the clinic] or what have you*’ (FM1).

Additionally, clinicians who had access to other aspects of the health system found it easier to work within the clinic, as they could access additional resources and support for their role. However, two participants reported that barriers to physical opportunity included a lack of equipment and funding, e.g., ‘*I think because the fact that the clinic’s not funded, I think that’s why, you know, there’s a limited time that we can do this*’ (HCP3). Finally, another commonly reported barrier to opportunity was a lack of communication between the clinic and other aspects of the siloed MND care system, ‘*there is no system for an urgent gastro[enterogolgist] referral. There is no system for any sort of procedural referral. So, you can’t refer for a PEG [percutaneous endoscopic gastrostomy], you have to refer for a consult and a PEG*’ (HCP4).

#### 3.1.3. Behavioural Determinants of Motivation to Be Involved in the Clinic

When participants discussed their motivation to participate in the clinic, it was clear that facilitators were over-represented. In terms of automatic motivation, participation in the clinic was reinforced by the perceived benefits it brought, both to plwMND and their carers.

*‘[It’s been a] positive experience because everyone’s very keen to make things better for the MND population. So [I’m] quite pleased to be sort of, I suppose, invited and to be asked to part of it’.*

(HCP8)

Additionally, participation was reinforced by using the clinic to streamline their patient care and easily access the expertise of other health professionals, e.g., ‘*[the MND-MDC] is good because we all get to discuss and know what’s going on, and make sure that he [plwMND] is being linked into the right services*’ (FM1). Similarly, most participants reported strong positive emotions about the impact of the clinic; for example, seeing the benefit of the clinic for plwMND and their carers gave participating clinicians a sense of pride and gratitude. ‘*The staff though were fantastic in integrating me in as quickly as possible… And I really appreciated that I got to speak to so many carers*’ (SCP1). Regarding reflective motivation, most participants were driven by positive beliefs about the consequences of the clinic for plwMND and their carers; however, some were unsure if the clinic could provide these benefits with its current limitations.

*‘So, I think for [plwMND] also that’s a really great opportunity for them to see everybody at the time. And what also works well is obviously having everybody together for that case conference to bring those ideas back’.*

(HCP6)

Participants who were plwMND were also motivated by a belief in the clinic staff’s capabilities, ‘*[the staff] are all good, you know what I mean? Nothing is a problem, they’re a great contact if I want them*’ (plwMND1). Finally, the motivation of all participants was commonly increased by formal strategies for behaviour regulation, such as standardised clinic information sheets and reminders, e.g., ‘*And we have to complete a sheet, so that each discipline has a section on the sheet where they can actually report some specific information which they use when we go to the case conference*’ (HCP3).

### 3.2. Results of the Inductive Analysis

The findings from the reflexive thematic analysis identified the impact and outcomes of implementing an MND MDC in a regional area.

Three main themes emerged from the interview data relating to the impact and outcomes: (Section 3.2.1) Implementing a ‘*good idea*’; (Section 3.2.2) ‘*Flushes out*’ local service gaps and/or challenges; and (Section 3.2.3) Positive outcomes. Table 2 summarises themes and sub-themes, supported by exemplar participant quotes.

#### 3.2.1. Implementing a ‘*Good Idea*’

*Identifying a service gap:* There was a ‘*need [for the MND-MDC], and there’s been a need there for a really long time actually*’ (SCP2). Participants reported that a holistic model of MND care was a priority that provided ‘*continuity*’, ‘*quality*’, and ‘*person-centred care*’ (HCP4,7) and was preferably designed as a ‘*one-stop shop*’ (SCP2), where all plwMND and their carers could be linked to ‘*the right people at the right time*’ (HCP3) whilst supporting equitable access to MND expert care, irrespective of age and funding scheme, as well as being ‘*closer to home*’, as prioritised by plwMND and family members.

*Engaging with stakeholders*: The neurologist acted as a ‘champion’ for the clinic by leading a mix of clinicians and non-clinicians on the Central Coast from across different sectors and organisations in initial discussions to explore locally driven solutions. This responsibility for the clinic included stakeholder engagement to be able to implement ‘a good idea’.

*Obtaining buy-in*: Buy-in was not easy even though a locally based MND MDC was deemed a ‘*good idea*’. The LHD could not offer formal support for the clinic’s running or provide the space. The lack of ‘*buy-in*’ by the LHD reduced the potential for service integration, limiting links to the public health workforce, preventing access to electronic medical records (EMRs), and restricting medical specialists’ involvement.

*Adoption of a good idea:* After further negotiation and the agreed use of a local private neurology clinic, it was a case of ‘*let’s just get this clinic up and running*’ (HCP3) and ‘*their [MND-MDC staff] ability and willingness to do it without any cost and charge*’ (HCP8). Ultimately, the lack of clinic resources limited intervention and implementation planning, as well as the frequency of the MND MDC to once every four months.

#### 3.2.2. ‘*Flushes Out*’ Local Service Gaps and/or Challenges

*Challenges accessing specialist care:* The MND MDC shed light on the many challenges in accessing expert care and support faced by plwMND living on the Central Coast, which resulted in some plwMND being described as lost to ‘*follow up after diagnosis*’ (HCP4). Despite plwMND highlighting the importance of ‘*access to people that know about it [MND]*’ (plwMND2), the cost was reported as ‘*a barrier for a lot of people that are on disability [pension] and that have and are no longer working*’ (SCP2). However, access to ‘*more research opportunities*’ (HCP4 and SCP2) and routine testing of respiratory function, for example, during research trials, was deemed by clinic staff as a motivator for plwMND to travel to metropolitan MND MDCs, highlighting the perceived importance of local routine monitoring and research opportunities.

*Lack of connected/coordinated care:* Navigating MND care and management was reported as complex and fragmented, with the lack of coordinated care affecting care experiences. The current local management of percutaneous endoscopic gastrostomy (PEG) feeding tubes was reported to often result in ‘*clients sit[ting] in ED for 11, 12 h waiting because they’re seen as a low priority waiting to get a PEG tube changed… to get a PEG tube changed is an absolute nightmare*’ (HCP6). Participants hoped that the MND MDC would provide greater opportunities to consult with harder-to-reach specialists, to ‘*have that instantaneous sort of response*’ (HPC5) and address care challenges. In reality, system silos limited these opportunities. *Care and support inequities*: Inequities in access to information, funding, and services post-MND-diagnosis were also described as a significant issue, particularly by plwMND.

*‘I [plwMND] actually was put off the NDIS for a long time. I should have got it a lot earlier. But I didn’t think that—I thought there were more deserving people of it than me but—and it turns out they’re going to get it anyhow’.*

(plwMND2)

Navigating the funding disparities of being older than 65, where ‘*the government pays some of it, but I pay the rest*’ (plwMND4), limited access to essential services and equipment, and for some participants, the MND-MDC was ‘*the only place I get overall management*’ (plwMND4).

*Challenges with information flow across care providers*: Participants hope that the MND MDC will improve information sharing between HCPs from across sectors and the MND MDC and that this would support more coordinated and shared-care practices and decision-making in the local community. However, information sharing was reported to rely on specific individuals, ‘*if that* [*information sharing*] *didn’t happen then there would be no feedback coming from Community Allied Health to the clinic and I guess* vice versa’ (HCP6). The role of the GP in care coordination was also unclear; for example, plwMND and carers stated they ‘*have never had any feedback* [*after the clinic*] *from their GPs*’, and GPs were perceived by a plwMND as ‘*a bit, sort of, in between, not knowing whether he’s looking after the case or the neurologist*’ (plwMND2).

#### 3.2.3. Positive Outcomes

*Filling service gaps*: The MND MDC was believed to demonstrate the value of cross-sector collaboration and a team-based approach in managing MND in regional areas. PlwMND, carers, and health and social care professionals reported that it provided a ‘*good* [*opportunity*] *to keep checking in*’ and ‘*people were listening*’ (FM2). It was believed that ‘*by talking to the staff you always learn*’ (plwMND3) about how to better manage the ongoing changes associated with living with MND.

*Raising awareness of services locally:* After attending the clinic, participants recognised that MND MDCs ‘*don’t have to be in cities. That we can actually develop smaller clinics*’ (HCP3). The MND MDC created an opportunity to routinely connect plwMND, carers, and clinicians in one location within their local area and at no out-of-pocket expense, reducing the risk of loss ‘*to follow up*’ (HCP4) and leading to being able to assess and respond to MND progression and refer or re-engage plwMND to local services and support, as well as providing solutions and advice to address specific challenges.

*Local community relationship building:* Despite the challenges of cross-sector working, bridging the public and private divide informally through the MND MDC meant health and social care providers were ‘*now connected with the hospital team so we can actually share that, those relationships; that’s really important*’ (SCP2).

*Informal cross-organisational and cross-sector working and connections*: These relationships facilitated information sharing; for example, the publicly funded health staff provided ‘*a little brief handover*(HPC5) to ensure that up-to-date information was available for the staff at the MND MDC. Greater links across organisations and between care providers were deemed a positive clinical outcome by the MND MDC staff:

*‘…the biggest plus is that we all now communicate and talk to each other about patients and maybe the relationships between people. Knowing that it’s not just solo practitioners out there dealing with MND patients’.*

(HCP8)

## 4. Discussion

The vision of the host state’s health service is to deliver ‘*a sustainable, equitable, and integrated health system delivering outcomes that matter most to patients and the community in regional, rural, and remote NSW*’ [18]. Considering this, we aimed to evaluate participant experiences of a regional MND MDC on the Central Coast, NSW, Australia, and explore the factors influencing MND MDC implementation. The TDF and COM-B were used to highlight the behavioural determinants requiring attention to overcome barriers to implementing an integrated model of care. Both the challenges and rewards of developing and implementing locally driven solutions within a complex health system to reduce existing care disparities and deliver evidence-based care for plwMND and their carers were explored.

Our study identified the complex (and frequently complicated) context in which multidisciplinary interventions must be implemented for plwMND. The expertise of HCPs in addressing the needs of individuals with MND was consistently perceived by plwMND and their families as valuable for engagement in the MND MDC [5,13,33]. Our findings concur with international research in MND multidisciplinary care [1] and are supported by findings in other studies in neurodegenerative disease care [56]. Previous studies revealed the benefits of targeting efforts to improve systemic and organisational knowledge and understanding to improve the integration of care [15,33,57,58]. Earlier studies also supported the need for stronger partnerships and connected care [36,57], as well as improved responsiveness to the needs of plwMND and families [35,59], while creating a more supportive and effective working environment for HCPs [59,60].

The current Australian context funding for disability and social care is largely based on age rather than disability (i.e., NDIS versus My Aged care) and has contributed to siloed clinical practices and delays in diagnosis and access to services, resulting in greater fragmentation and inequities in service and support provision [24,25]. Further, the poor connectivity of disability care to hospital, primary, and community care has created a chasm that is even harder to bridge. Locally based solutions designed around specific contexts and communities, such as the regionally based MND MDC, can help overcome these barriers to create timely, coordinated, and equitable access to services. However, despite the potential value and growing demand for the MND MDC, the lack of funding and the legislative restrictions on public health employees in supporting and participating in a private clinic challenged the delivery of the MND MDC, as well as future planning for the adoption and sustainability of the MND MDC. As highlighted by Schroeder et al. (2021), “different funding models create different financial incentives, which in turn lead to different services being offered and accessed. Quite simply, funding models can impact access to healthcare, and therefore health outcomes, in a substantial way” [61] (p. 2).

While adapting and embedding the MND MDC into existing care practices would support MND management across the local area, it cannot be achieved without first developing the necessary support infrastructure across the local health system. In this study, staff at the MND MDC believed that relocating the MND MDC to sit within the public hospital system would be beneficial for connecting with specialists and increasing access to facilities. This highlighted the importance of the clinic’s location and governance structure, not only to building a capable and more sustainable workforce and improve access to specialists and specific facilities but also to support greater cooperation, collaboration, and partnership across the health system, which is necessary for delivering models of integrated care [29]. This collectively could empower formal and informal networking across organisations, which are all important for the spread, scale-up, and sustainability of new interventions [62]. Similar to previous investigations, our study has emphasised the influence of contextual factors in the implementation of health system change, and the necessity to account for this influence in creating and supporting sustainable change [44,63].

Despite these barriers, our study resonated with Walls et al. 2024 and described the protective impact of having the right clinicians in an MND MDC to optimise their role and provide the specialist care required by plwMND and their carers [60]. For example, existing trusting connections between clinic staff allowed for the development of collaborative practices, interdisciplinary working, and the team’s capabilities in delivering a low-complexity clinic, as well as the ability to trial the implementation of an MND MDC in a regional area [64]. Additionally, most clinic staff had high levels of knowledge of the health and social system that they had previously developed from working across silos or in different parts of the health system, which was a strong enabler in overcoming the identified system barriers. Local network building with representatives from across sectors, such as the health, disability, aged-care, and not-for-profit sectors, was important [36] and ensured that the MND MDC adopted an integrated and person-centred approach. These results emphasise the importance of carefully choosing a core of locally experienced and knowledgeable staff for future regionally led MND MDCs.

Our results also demonstrated the importance of the champion role [65] in facilitating the adoption and implementation of the MND MDC and obtaining allied health and medical ‘buy-in’. This research concurs with Bonawitz et al. (2020) that the champion role is not just ‘*what* they do but also in *who* they are’ [65] (p. 9). The clinic neurologist acted as the MND MDC champion, providing leadership and taking personal responsibility for clinic’s success and operation despite the competing demands on their time. They demonstrated a strong commitment to plwMND and their families, even with the lack of resources to fund the MND MDC, which the MND-MDC staff acknowledged, felt motivated by, and were keen to support and nurture, even developing clinic processes and workarounds to overcome sector silos.

The demand for the MND MDC clinic grew over the two years of its evaluation and has subsequently exceeded capacity, with clinics extending over the whole day. This growth demonstrated a need for the service but funding limitations and competing demands on the MND MDC staff, who all had other, paid work commitments, reduced their capacity to attend. However, despite these challenges, the staff demonstrated their ability to function as an interdisciplinary team based on a good understanding of their roles, effective communication, a strong team culture, including positive relationships, trust, and commitment, flexibility to cover roles, and the shared belief in the purpose of the clinic, which are all important principles for effective transdisciplinary working, as reported by Nancarrow et al. (2013) [66]. In addition, the MND MDC’s strong trusting relationships with plwMND and their family members contributed to their sense of value and motivation to make suggestions about improvements to the clinic, leading to the inclusion of a carer support representative after the clinic had been running for 18 months [67]. Together, these strategies all contributed to the positive outcomes of the MND MDC.

Despite this positivity, there were early warning signs of burnout in our data. For example, negative emotions, frustrations, the clinic’s lack of goals, and limited opportunities to improve the efficiency of the MND MDC were highlighted in the TDF and COM-B analyses. Interventions to address these determinants could be developed using the Expert Recommendations for implementing Change (ERIC) [68,69], a useful tool that provides a foundation for constructing multidimensional intervention strategies to improve outcomes at both the individual and organisational levels [69]. Such strategies could include providing MND-specific education and training to HCPs and SCPs across sectors, which would upskill the entire workforce regarding the care of MND, promote the role of the MND MDC within the health system, and support HCPs working with MND [60]. Strategies to restructure the clinic’s environmental context could include sourcing funding, resource and information sharing, network weaving, formally clarifying different professional roles, developing leaders, increasing engagement with plwMND and carers and developing shared goals and decision-making.

## 5. Limitations

As this study only involved people who had pre-existing knowledge of the MND MDC, it would have been valuable to obtain a more comprehensive representation of MND referral and care pathways by describing the experiences of other HCPs and SCPs working with plwMND across all health and social care sectors on the Central Coast. Despite the small number of plwMND and family carers participating, the SAG provided guidance and validation of the data analysis to ensure the accuracy of the results. The researchers were aware of the potentially positive response bias, particularly from plwMND and family carers, and took steps to mitigate this through building rapport, using open questions, and ensuring anonymity.

## 6. Conclusions

This study demonstrates the cross-sector commitment to providing equitable access to an MND MDC in a regional area to optimise timely, person-centred, and integrated care for plwMND and their families. These specialist clinics must overcome complex organisational boundaries and age-related service funding models to deliver connected and coordinated care across the continuum, create locally driven solutions, and build strong cross-sector and organisational partnerships. The evaluation of the MND MDC demonstrated the need to address behavioural determinants to practice change through co-creation with a range of stakeholders and targeted multidimensional intervention strategies to improve outcomes at both the individual and organisational levels. This study highlights the need to co-design solutions to overcome complex sector and organisational boundaries to optimise care pathways, collaborative practices, partnership, and communication, which will guide integrated MND care initiatives.

## Figures and Tables

**Table 2:** Summary of themes and sub-themes.

Themes and Sub-Themes	Exemplar Quotes
3.2.1 Implementing a ‘*good idea*’	
➢ Identifying a service gap	*‘[before the clinic] there wasn’t really continuity* [of care] *there was no ownership [MND management]. There wasn’t even necessarily any follow up after [MND] diagnosis’.* (HCP4)
➢ Engaging with stakeholders	*‘[before the clinic] we [MND NSW] were talking for some time about organising, yes, about really thinking that there’s a need to have the clinic on the Central Coast. And linking in with people that we knew had a passion’.* (SCP2)
➢ Obtaining ‘buy-in’	*‘I certainly have that support [from the public health system] that I can attend the clinic in a private capacity’.* (HCP5)
➢ Adoption of the ‘good idea’	*‘[The MND-MDC is] A positive experience, because everyone’s very keen to make things better for the MND population. So quite pleased to be… invited and to be asked to be part of it’.* (HCP8)
3.2.2 ‘*Flushes out*’ local service gaps and/or challenges	
❖ Challenges accessing specialist care	*‘In most of those situations the [other MND-MDCs around NSW], you still are paying privately for the clinic. In a way that makes it a niche…, it does actually disregard the rest of that population’.* (SCP2)
❖ Lack of connected/coordinated care	*‘Having a PEG nurse in the community or even just in the hospital that patients could book into a clinic to go and have a tube changed would be amazing. Just to have a person to link in with would be amazing’. (HCP6)*
❖ Care and support inequities	*‘Look, to be very, very honest, I couldn’t afford to go and see [a neurologist]. So having access to [neurologist at MND-MDC] that knew about it [MND] in that way was fantastic’.* (plwMND2)
❖ Challenges to information flow across care providers	*‘There’s lots of people’s assumptions about how and what we [in speciality] do, but not everyone really knows what we can do’.* (HCP8) *‘no discussions with the GP about information obtained from MND-MDC’.* (plwMND3)
3.2.3 Positive outcomes	
○ Filling service gaps	*‘I think particularly for clients who are in the earlier stages of disease or are slow progressing who are not getting a lot of input outside of the clinic, I think for them it’s a really useful time for them to come in every three or four months, attend the clinic, see everybody that they need to see, have that check in, are there any issues and then they go off on their merry way until the next clinic in another four months’ time. So, it’s a really great way to still capture those clients and they don’t get lost to follow up because there’s no current issues for them’*. (HCP6) *‘I think it’s good that the carers’ [Support Unit representative] are there [at the MND-MDC] and I can reach out to them’. (FM1)*
○ Raising awareness of services locally	*‘I think something that I didn’t anticipate at the beginning [of the clinic] was the need for people who knew how the community support services worked. And that’s where the MND guys [MND Support Association] have come in… They’ve been absolutely integral in the day to day outside clinic, like real life problem solving’*. (HCP4)
○ Local community relationship building	*‘I think for us here on the Central Coast, we have people in the community with the experience. And I think that’s the difference with the clinic up here [compared to Sydney clinics] in that we already have that great [community] service available’*. (HCP6)
○ Informal cross-organisational and cross-sector working and connections	*‘It’s working as a team, hands down it doesn’t matter what body, organisation, bucket of funding, whether it’s health, whether it’s community team, whether it’s NDIS, you’re still working for the same cause, and you’re still working towards better outcomes for that participant’.* (SCP2)

**Table 3:** Health and social care participant characteristics.

Professional Role and Employment Sector	Number (*n* = 8)
Healthcare professionals (*n* = 6)	
Private and public health sectors	3
Public health sector only	1
Private health sector only	2
Social care professionals (*n* = 2)	
Public health sector only	1
Not-for-profit sector only	1

**Table 4:** People living with MND and family carer characteristics.

MND MDC Attendees	Number (*n* = 6)	Age	Gender	Work Status
People living with MND (*n* = 4) and carers (*n* = 2)				
People living with MND	3 1	≤64 years ≥65 years	Male Male	Unemployed Retired
Carers	1 1	≤64 years ≥65 years	Female (spouse) Female (spouse)	Employed Retired

## Data Availability

Due to the nature of this research, the participants of this study did not agree for their data to be shared publicly; therefore, supporting data are not available.

## Supplementary Materials

The following supporting information can be downloaded at: https://www.mdpi.com/article/10.3390/healthcare13070801/s1, A. MND-MDC staff Interview Guide; B. People living with MND and their family members accessing the MND MDC interview guide.
