# Supplementary material for: Connecting Care Closer to Home: Evaluation of a Regional Motor Neurone Disease Multidisciplinary Clinic
Source: Healthcare (Basel). 2025 Apr 2;13(7):801. doi: 10.3390/healthcare13070801 (PMC11988506; doi:10.3390/healthcare13070801)
Supplement: Supplementary file 1 [file healthcare-13-00801-s001.zip › healthcare-3521055-supplementary.pdf]

Supplementary Material

A. **MND-MDC staff Interview Guide**

Participants will be interviewed once only.

These questions are a guide only. A trained interviewer will undertake the interviews and will ensure there is room for free discussion.

Introductions and preamble

Thank you, <name> for agreeing to speak with me today.

You would have read in the study information sheet that we will record this session, but all transcripts will be de-identified, and you will not be named in any reports, and we will seek your permission to use any direct quotes. You can choose to stop this interview at any time if you wish without giving a reason.

Do you have any questions before we start?

So, if you are happy to go ahead, we will get started – is that OK with you?

Questions here are prompts for what may be applicable to the interviewee. Often interviewees will cover questions on the list without being specifically asked – avoid repeating what was already covered.

1. Tell me about how the clinic runs and your role in the clinic and with people living with MND?
2. Has your role changed over the implementation of the MND MDC in any way?
a. If so, tell me about why your role changed?
2. Who are the key people that make the MND MDC possible, and why?
3. Which components of the service do you believe have worked the best since the implementation of the MND MDC? (Tell me why)
4. Do you think that the service was implemented as you expected from the beginning or did changes have to be made over time? (If so, what changes and why were these needed?)
5. Tell me about how MND MDC fits in with the broader MND services in your region? Consider health, NDIS, aged care and not for profit sectors.
6. Apart from the key people involved in delivering the service, have other clinical staff become involved over time? Have they linked their patients with MND MDC?
7. What role has telehealth played in delivering the service?
8. Were there any unexpected outcomes and consequences of implementing MND MDC in your region, both positive and negative?

i. What factors helped in mitigating for unintended negative outcomes (if any)?

9. Thinking back about implementing the MND MDC, what were the main barriers? How were these overcome? If not overcome, do you have any ideas what could be done to overcome the barriers?
10. Change in the health system is often difficult – what factors facilitated implementation of MND MDC in your region?
11. What support did you get from CCLHD? What else would have been helpful from the project team when implementing MND MDC?
12. Thinking back since the implementation of the clinic what would you have done differently to implement MND MDC in your region?
13. Do you believe that MND MDC has kept up with demand for the service?
14. When you think about delivering MND services in your region over next three to five years, what role will the MND MDC play?
15. Are there any specific plans to keep the service going, if so, what are they? How do you think this could be achieved?

B. People living with MND and their family members accessing the MND MDC interview guide

People living with MND, and their family members will be interviewed once. Interviews will be conducted separately, if possible, but can be done together if preferable.
These questions are a guide only. A trained interviewer will undertake the interviews and will ensure everything is set up in such a way that will make the interview as easy as possible for you.

Introductions and preamble

Thank you, <name> for agreeing to speak with me today.
You would have read in the study information sheet that we will record this session, but all transcripts will be deidentified and you will not be named in any reports, and we will seek your permission to use any direct quotes. You can choose to stop this interview at any time if you wish without giving a reason.
Do you have any questions before we start?
So, if you are happy to go ahead, we will get started – is that OK with you?

---

1. Tell me a little bit about how MND is impacting you/ your family members? For how long have you/ they had this condition? What health and social care services and support do you/they regularly need and use?
2. Can you tell me how these services and health care professionals such as GP, Physio, OT currently work together with you to help manage your/their MND? How do they communicate with you and each other?
3. How did you find out about the MND MDC, and can you tell me how you/they were referred to the MND MDC?
4. What was involved in helping to make your decision to attend the clinic? Did you chat with someone, receive any written information etc? How many clinics have you attended?
5. Since you have already attended at least one clinic, can you describe your experiences of the clinic, tell me more about the type of information, guidance, help the staff provided at the clinic and do you think the staff at the clinic addressed your/their specific needs and/ or goals?
6. What do you think to the whole set of the clinic such as the location, resources available, payment process, disability access, timing, usefulness, knowledge and skill of staff to help manage life with MND?
7. Can you tell me what information you received after the clinic and if there were any changes made such as referrals to specialists, new equipment, more discussions with GP etc after the clinic?
8. Have you been to a MND MDC that is based in the city such as Sydney and Newcastle before or is this your first experience of a MND clinic? Can you tell me more about how your experiences compare?
9. In what way, if any, does the MND clinic contribute to your overall management of MND?
10. Do you feel having a MND clinic located closer to home is important to you?
11. What do you feel is important to the overall effectiveness of the clinic?
12. We would really value any additional thoughts or experiences that would help us improve the running and effectiveness of the clinic.
